# Extraction of consensus protein patterns in regions containing non-proline *cis *peptide bonds and their functional assessment

**DOI:** 10.1186/1471-2105-12-142

**Published:** 2011-05-10

**Authors:** Konstantinos P Exarchos, Themis P Exarchos, Georgios Rigas, Costas Papaloukas, Dimitrios I Fotiadis

**Affiliations:** 1 Unit of Medical Technology and Intelligent Information Systems, Dept. of Materials Science and Engineering, University of Ioannina, GR 45110, Ioannina, Greece; 2 Institute of Biomedical Technology, CERETETH, GR 38500, Larissa, Greece; 3 Dept. of Medical Physics, Medical School, University of Ioannina, GR 45110, Ioannina, Greece; 4 Biomedical Research Institute, Foundation for Research and Technology-Hellas, University of Ioannina, GR 45110, Ioannina, Greece; 5 Dept. of Biological Applications and Technology, University of Ioannina, GR 45110, Ioannina, Greece

## Abstract

**Background:**

In peptides and proteins, only a small percentile of peptide bonds adopts the *cis *configuration. Especially in the case of amide peptide bonds, the amount of *cis *conformations is quite limited thus hampering systematic studies, until recently. However, lately the emerging population of databases with more 3D structures of proteins has produced a considerable number of sequences containing non-proline *cis *formations (*cis*-nonPro).

**Results:**

In our work, we extract regular expression-type patterns that are descriptive of regions surrounding the *cis*-nonPro formations. For this purpose, three types of pattern discovery are performed: i) exact pattern discovery, ii) pattern discovery using a chemical equivalency set, and iii) pattern discovery using a structural equivalency set. Afterwards, using each pattern as predicate, we search the Eukaryotic Linear Motif (ELM) resource to identify potential functional implications of regions with *cis*-nonPro peptide bonds. The patterns extracted from each type of pattern discovery are further employed, in order to formulate a pattern-based classifier, which is used to discriminate between *cis*-nonPro and *trans*-nonPro formations.

**Conclusions:**

In terms of functional implications, we observe a significant association of *cis*-nonPro peptide bonds towards ligand/binding functionalities. As for the pattern-based classification scheme, the highest results were obtained using the structural equivalency set, which yielded 70% accuracy, 77% sensitivity and 63% specificity.

## Background

**Figure 1:**
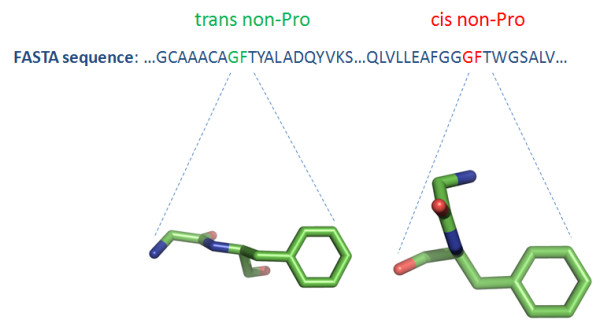
**Conformational isomers of a Glycine-Phenylalanine peptide bond detected in the beta-ketoacyl-acyl carrier protein synthase III (PDB id: **1HNJ**)**.

Peptide bonds occur predominantly in the *trans *conformation; only a small fraction adopts the energetically less favored *cis *conformation [1]. *Cis *peptide bonds are further distributed in two categories, according to the residues they connect; namely, imide bonds which occur between any amino acid and proline, and amide bonds which bind any amino acid and any amino acid except proline, out of which 5.2% and 0.03% are in *cis *conformation, respectively. It should be noted that there is a significant association between the resolution that a protein structure has been solved and the number of *cis *peptide bonds detected [1]. Consequently, the exploration of *cis *peptide bonds and especially the amide ones was hampered for several years due to the limited amount of high quality 3D protein structures. However, an increasing amount of protein molecules solved at high resolution has recently facilitated more systematic studies. Moreover, *cis*-nonPro peptide bonds are actually found to occur more frequently than previously thought and are often located at or near functionally important regions of the proteins, such as active sites [2] and dimerization interfaces [3]; these facts triggered further studies in order to unravel the molecular mechanism of these rare but highly significant configurations of the peptide bonds (Figure 1).

The configuration of the peptide bond is inherently affected by the surrounding residues, therefore *cis*/*trans *isomerization is encoded to some extent in the primary amino acid sequence; as well as from residues that are located close in space but necessarily in sequence. Nuclear Magnetic Resonance (NMR) experiments on oligopeptides have proven that the peptide bond between two amino acids is influenced by the sequence which spans the proximity of the bond [4]. This is in accordance with several methods aiming to predict the peptide bond conformation using only the amino acid sequence, or sequence-extracted features [5-9]. These methods exploit information extracted from the residues adjacent to the peptide bond in order to predict mainly the conformation of imide bonds, however, in [8] and [9] they were able to predict the peptide bond conformation between any two amino acids. More specifically, Pahlke *et al*. [8,10] developed an algorithm based on an extension of Chou-Fasman parameters and derived four rules to predict conformation of the peptide bond by taking into account only the secondary structure of amino acid triplets. Exarchos *et al*. [9] utilized a large number of sequence extracted features fed into an SVM classifier coupled with a feature selection algorithm to predict the peptide bond conformation between any two amino acids. Although, the identification of the peptide bond conformation solely from the primary amino acid sequence is of great importance, the black-box architecture of these methods does not provide adequate (if not any) justification about the respective predictions; hence limited biological insight can be deduced. Towards the exploration of similar sequence-driven characteristics of the protein structure (e.g. protein disorder [11] and secondary structure formations [12,13]) or even for proline *cis *peptide bonds [14], pattern-based methodologies have been proven quite beneficial.

In this work, we analyze regions containing *cis*-nonPro peptide bonds in order to detect regular expression-type patterns which are associated with these regions. Initially, we discover all patterns in the vicinity of *cis*-nonPro peptide bonds; patterns are extracted using exact pattern discovery as well as considering certain conservative substitutions with biological insight among the amino acids, specifically with respect to chemical and structural equivalencies. Next, we assess the representation of the extracted patterns in order to omit redundant patterns and come down to a list of patterns that have high coverage of *cis*-nonPro regions and low false discovery rate (i.e. matches with *trans*-nonPro regions). The retained patterns are further used to formulate a pattern-based classifier which discriminates between *cis*-nonPro and *trans*-nonPro peptide bonds. Finally, we compare the retained associations with the ELM [15] functional repository in order to rediscover known functional implications of *cis*-nonPro peptide bonds but also identify potentially novel ones.

## Methods

**Figure 2:**
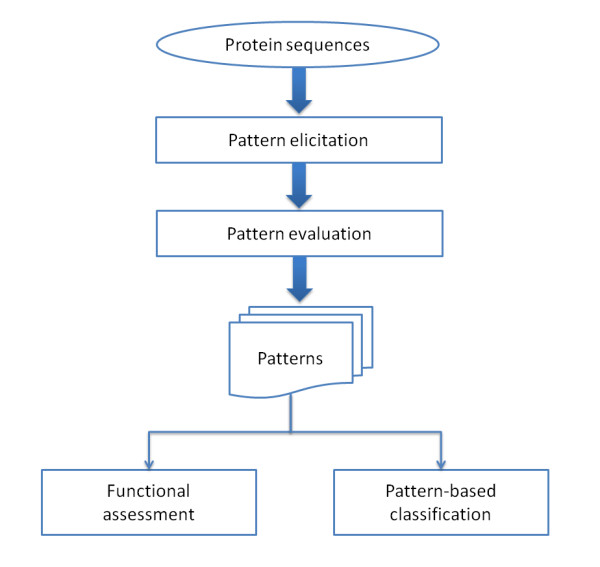
**Overview of the employed methodological analysis**.

The overview of the proposed three-stage methodology is depicted in Figure 2. In the sections which follow we describe the constitution of the employed datasets, next the proposed methodology is presented in detail and subsequently the procedure for functional assessment is shown.

### Dataset

The reference dataset employed in the current study has been extracted from the Protein Data Bank [16]. More specifically, 3050 high quality protein structures have been selected that comply with the following criteria: structure determination using X-ray crystallography with a resolution better than 2.0 Å, sequence identity less than 25% and R-factor less than 0.25. The annotation was performed using the VADAR (Volume Area Dihedral Angle Reporter) software [17] where bonds with dihedral angles within the range of ± 30° were classified as *cis *and bonds with angles between 150° and 210° were classified as *trans*.

**Figure 3:**
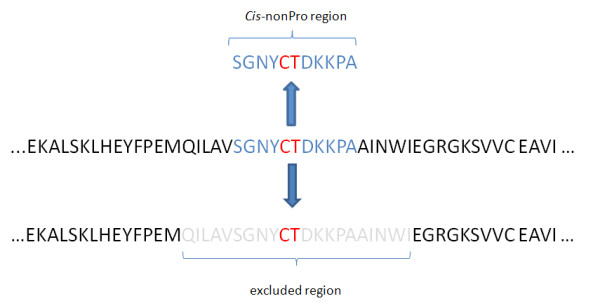
**Construction of the *CNP *and *TNP *datasets**.

For each *cis*-nonPro peptide bond in the above protein sequences, we assembled a window of neighboring residues (± 5) which influences significantly the peptide bond conformation [7,9]. In a similar manner we formulated a region of residues surrounding *trans*-nonPro peptide bonds, however, it should be noted that the immediate ± 5 *trans*-nonPro residues were excluded to avoid interclass overlapping regions, i.e. the regions of amino acids shared by a *cis *peptide bond and adjacent *trans *bonds (Figure 3). Thus, we formulate two sets of amino acid sequence segments, where the length of each segment is 11 residues. If a segment contains a *cis *peptide bond in the center, it is assigned in a dataset called hereafter *CNP *(*cis*-nonPro), otherwise it is assigned in the *TNP *(*trans*-nonPro) dataset. The *CNP *and *TNP *datasets contain 318 and 685716 sequence regions, respectively. The first and last five residues from every protein sequence were also excluded from our study since they do not have enough neighboring amino acids to constitute the required 11-length segment.

### Pattern elicitation

The list of regions contained in the *CNP *dataset, is provided as input to the TEIRESIAS pattern discovery algorithm [18] in order to detect frequent patterns in the neighborhood of *cis*-nonPro bonds. During pattern discovery all provided regions are searched for elementary patterns exceeding a minimum support threshold, which are being progressively combined into larger patterns. All extracted patterns are guaranteed to be maximal, i.e. they cannot be made more specific without simultaneously affecting their length and/or composition, conforming to a set of user specified criteria. Specifically, the maximum length of the extracted patterns is set to 11 residues (W = 11), where at least three literal characters (i.e. non-wild-characters) are required (L = 3), and every pattern must have minimum support K = 2. The maximum length is justified by the length of all segments, which is 11, and the minimum number of literals is dictated by the convergence requirements of the TEIRESIAS algorithm. Moreover, patterns with fewer literal characters would merely yield quite generic and nonspecific patterns. In the current study three types of pattern discovery are performed: i) exact pattern discovery, ii) pattern discovery using a chemical equivalency set ([AG], [DE], [FY], [KR], [ILMV], [QN], [ST]) and iii) pattern discovery employing a structural equivalency set ([CS], [DLN], [EQ], [FHWY], [ITV], [KMR]). Residues enclosed in square brackets ("[ ]") form a character class and are considered identical during the pattern discovery procedure.

The employment of substitution groups during pattern discovery is likely to uncover underlying patterns present in regions containing *cis*-nonPro peptide bonds. For example a position occupied in the sequences under consideration almost with the same frequency by a phenylalanine, a histidine, a tryptophan or a tyrosine, will be assigned to the [FHWY] group, thus indicating a tendency towards aromatic amino acids. Hence, the incorporation of these biologically inspired equivalency sets might provide adequate reasoning and insight into the molecular basis of *cis*-nonPro formations.

### Pattern evaluation

After the initial elicitation of patterns from *cis*-nonPro regions, each extracted pattern is evaluated to quantify how generic it is by comparing it against major biological datasets and representative negative control sets. This is due to the fact that the extracted patterns have been derived from a single-class dataset (i.e. *CNP*), leading to questionable selectivity that needs to be further validated. Specifically, for each extracted pattern the log-likelihood is calculated using the "*evaluate3plets*" module in TEIRESIAS, representing the likelihood of the extracted pattern occurring by chance [19]. The background model used by TEIRESIAS to estimate the significance of a pattern is based on a second-order Markov model of protein sequences in the GenPept database [19,20]. Moreover, it is very important to measure the representation of the extracted patterns in the negative control set, the *TNP *dataset; therefore, using each pattern as a predicate, we search in regions containing *trans*-nonPro peptide bonds for potential matches. Afterwards, a score is attached to every pattern, which is based on the proportional representation of the pattern under consideration in the *CNP *and the *TNP *dataset. Assuming that *P *is a pattern and *M(P) *is the set of regions matching *P*, the following scoring function is defined:(1)

where the *norm *= |*CNP*|/|*TNP*| is employed as a normalizing factor, since the representation of a pattern in every dataset is expected to be approximately proportional to the size of each dataset. Using the aforementioned methodology we are able to efficiently phase out the class imbalance in our dataset, without screening potentially valuable and informative negative examples. If we employ sampling-based approaches we might come across certain drawbacks; in the case of undersampling we randomly choose a subsample of regions from the *TNP *dataset, thus imposing bias on the choice of the samples but also leading to a considerable reduction in the available negative samples, consequently resulting to great loss of information. As for oversampling the instances of the minority class, this would lead to a significant increase of repetition in the resulting dataset, amplifying at the same time potentially noisy samples that might be present in the *CNP *dataset.

So far the extracted patterns have been evaluated in terms of how generic they are as well as how they differentiate from *trans*-nonPro regions; another issue raised is that some of the extracted patterns might be partially overlapping, therefore retrieving intersecting sets of regions. In order to remove overlapping patterns of this kind, first, we sort the patterns according to the *sc_score *obtained from the previous step and, next, we use each pattern in turn to search for matches in the *CNP *and *TNP *dataset. The outcome is a set of patterns with 1-N relationship with the regions, meaning that each pattern retrieves several regions and each region is retrieved by no more than one pattern. The algorithm terminates either when 100% coverage of *cis *regions is achieved or if all patterns have been enumerated. Our aim is to come down to a list of patterns that achieve high coverage of the *CNP *regions and at the same time retrieve as few regions as possible from the *TNP *dataset (i.e. False Discovery Rate - FDR). The above steps are followed for all sets of patterns, extracted using each type of pattern discovery.

All the above steps are motivated by the scarcity of *cis*-nonPro conformations as well as their ambiguous nature in terms of residues' composition. An extra burden is also posed by the overwhelming amount of *trans*-nonPro formations in protein sequences. However, the sequence of actions described above reduces the possibility of maintaining redundant patterns, as it will be shown in the following.

### Functional assessment

The retained patterns are then compared against the ELM resource in order to identify propensities towards certain biological functions. ELM is a curated repository containing experimentally validated protein patterns along with their respective functionality; moreover, for each deposited pattern one or more Gene Ontology (GO) [21] terms are assigned. For the pattern-pattern comparison the CompariMotif algorithm [22] is employed which quantifies similarity between two patterns based on shared information content. Initially, exact matches between patterns are sought; if no precise matches are found and the patterns under consideration share enough common residues, a sliding window comparison is performed scoring all possible overlaps between the patterns. All reported non-random associations in the output of CompariMotif are assigned a score, called hereafter *CM_score*, which denotes the similarity between two patterns and takes into account the patterns' overlap, length and degeneracy. Moreover, in order to ascertain that the reported associations are non-random and designate statistically significant functional propensities, we perform a chi-square test between the observed frequencies assigned by Comparimotif and the frequencies expected by chance. The expected frequencies are calculated based on the representation of each functional class within ELM.

### Pattern-based classification

The next step of our methodological analysis involves the exploitation of the extracted patterns for predicting the *cis*/*trans *isomerization of amide peptide bonds. In order to evaluate the predictive potential of the extracted patterns we utilize the following procedure. We randomly split the *CNP *dataset, thereby assembling a training set of 225 *cis*-nonPro regions (i.e. 2/3 of the *CNP *dataset), and using the remaining 93 regions (i.e. 1/3 of the *CNP *dataset) for testing. The regions of the training set are subject to the aforementioned steps of the proposed methodological analysis, i.e. pattern elicitation using three types of pattern discovery and pattern evaluation. Specifically for the latter step (pattern evaluation), the control group is assembled by randomly sampling without resubstitution an equal number of *trans*-nonPro regions from the *TNP *dataset. The retained patterns from each type of pattern discovery are fed as input to an algorithm which searches across the testing set, and if a match is found the respective region is marked as *cis*, otherwise, if no pattern matches the region, it is marked as *trans*. The testing set is comprised of 93 *cis*-nonPro regions and an equal-sized randomly assembled subset of the *TNP *dataset. Especially for the *trans*-nonPro regions comprising the testing set, we performed random sampling 5 times, resulting in 5 testing sets [23]. The predictive potential of the patterns identified in the training set, is subsequently quantified against 5 independent testing sets, and the results are averaged, in order to gain a more reliable overall assessment.

It should be noted that the permutations of the *CNP *and *TNP *datasets are performed only in this step, in order to assess independently the performance of the pattern-based classification scheme. The list of patterns reported throughout the manuscript involved the entire *CNP *dataset, in order to get the most out of the initial set of available *cis*-nonPro peptide bonds.

## Results & Discussion

The aforementioned methodological analysis has resulted in a list of patterns that are indicative of regions containing a *cis*-nonPro formation. The extraction of patterns describing these regions is very important due to the scarcity of *cis*-nonPro peptide bonds; therefore, the elicitation of consensus patterns extrapolates the knowledge that can be gained from a limited amount of data that is currently available.

### Amino acid patterns

Initially, after providing all the *cis*-nonPro regions as input to the TEIRESIAS algorithm, we extracted 4815 patterns using exact pattern discovery, whereas the employment of equivalency sets yielded 38904 and 32812 patterns when chemical and structural equivalencies were considered, respectively. Using eq. 1 the *sc_score *is calculated and attached to all extracted patterns; clearly *sc_score *ranges between 0 and 1 with values above 0.5 indicating propensity of a pattern towards *cis *configurations. However, as we are interested in patterns with a strong preponderance for *cis *regions we set a threshold of 0.90, thus discarding patterns that have high representation in the *trans *regions. Subsequently, the retained patterns are significantly reduced, specifically 1622 patterns are maintained when no equivalency set is employed, 8251 for the case of chemical equivalency set and 7347 with the structural equivalency set. Afterwards, we aim to remove redundant patterns, so that eventually each region is matched by at most one pattern, i.e. patterns that match more or less with the same regions are scrutinized; the resulting set must ensure high coverage of the *cis *regions and at the same time low False Discovery Rate (FDR). As mentioned in the previous section, for this purpose the patterns are sorted according to the *sc_score *which is a proportional indicator of *cis *and *trans *matches. An overview of the patterns maintained using all types of pattern discovery, after each filtering step as well as the respective coverage and FDR metrics is shown in Table 1. No metrics are provided for the second column as it is an unsorted superset of the last column.

**Table 1:** Overview of patterns maintained after each preprocessing step and for all types of pattern discovery.

Exact pattern discovery			
	***TEIRESIAS***	***sc_score > 0.90***	***Non-redundant***

***Number of patterns***	4815	1622	231

***Coverage (%)***	100%	-	100

***FDR (%)***	3.58	-	0.25

**Chemical equivalency set**			

	***TEIRESIAS***	***sc_score > 0.90***	***Non-redundant***

***Number of patterns***	38904	8251	235

***Coverage***	100	-	100

***FDR***	6.79	-	0.03

**Structural equivalency set**			

	***TEIRESIAS***	***sc_score > 0.90***	***Non-redundant***

***Number of patterns***	32812	7347	225

***Coverage***	100	-	100

***FDR***	6.69	-	0.02

We observe that even though high coverage values can be achieved both by the patterns initially extracted from TEIRESIAS, without employing any of the proposed preprocessing steps, and by the non-redundant set of patterns, FDR is quite high in the former case, especially given the large number of *trans*-nonPro peptide bonds (6.79% equals to 46560 *trans*-nonPro bonds). For the non-redundant set of patterns, FDR is kept in all types of pattern discovery below 1% ensuring low association of the retained patterns with *trans*-nonPro regions, while keeping the list of descriptive patterns relatively refined. Therefore, the proposed methodology could be employed as a basic preprocessing step towards the discrimination between *cis*-nonPro and *trans*-nonPro formations, as it is able to reduce the initial input of sequences by more than 99% and at the same time to maintain all the *cis*-nonPro regions of interest intact.

As it is shown in Table 1 the non-redundant set for all types of pattern discovery contains around 200 patterns. The relatively high number of retained patterns is partially attributed to the fact that we wanted to formulate a dataset able to achieve complete coverage of *cis *formations keeping at the same time false positives at a very low rate. Smaller sets of more generic patterns could be extracted, however, this could lead to much higher FDR values. The total number of retained associations is slightly less important than keeping patterns that precisely describe regions around *cis*-nonPro bonds and discriminate them from regions with *trans*-nonPro bonds.

Table 2 contains an indicative subset of the highest scoring patterns extracted using each type of pattern discovery. The complete list of patterns is accessible via the web due to space limitations. The patterns have been sorted according to the *sc_score *metric. In addition, the log-likelihood of each pattern is shown, determining how generic each pattern is; we observe that for all reported patterns the log-likelihood is very low, denoting that the patterns under consideration are highly unlikely to occur by chance.

**Table 2:** The 20 highest scoring patterns sorted in descending order by *sc_score*.

Exact pattern discovery			Chemical equivalency set			Structural equivalency set		
**Pattern**	**sc_score**	**Log-likelihood**	**Pattern**	**sc_score**	**Log-likelihood**	**Pattern**	**sc_score**	**Log-likelihood**

KPGKGRRK	1	-37.67	KPGKGRRK	1	-37.67	KPGKGRRK	1	-37.67

EDGTKEPLL	1	-42.25	G.[AG][DE].K..SL	1	-23.91	S.S[ITV]H..N	1	-19.73

HAESGEYGL	1	-44.51	[ILMV][ILMV].[AG].D.AT	1	-21.70	E.V[DLN].[KMR]P	1	-18.28

LGTVINQL	1	-36.61	G[AG].[DE][ILMV]K.[ILMV]S[ILMV]	1	-31.33	MLQ...[ITV].[KMR]	1	-19.03

ADEAT	1	-20.01	G..[FY]W[QN]..D[ST]	1	-25.89	[EQ].GYT.R	1	-20.32

ALNALKLVT	1	-41.86	LGTVINQL	1	-36.61	[KMR]..QGY..R	1	-20.23

YFT...I	1	-14.57	EDGTKEPLL	1	-42.25	EDGTKEPLL	1	-42.25

CLA..VN	1	-20.96	GA.D[DE]A[ST]	1	-24.88	LGTVINQL	1	-36.61

R..DP....VV	1	-20.01	ADEAT	1	-20.01	HAESGEYGL	1	-44.51

H.YSQ	1	-15.76	[ST]..A[DE]G.A	1	-18.38	ADEAT	1	-20.01

VYL..L...Y	1	-20.29	[AG][ILMV]..L[KR]L..D	1	-20.11	ALNALKLVT	1	-41.86

NAW..D	1	-15.89	T.R.E..A.[ILMV]	1	-18.48	GG...[KMR]M..L	1	-19.48

A...KHF.G.G	1	-26.56	[ST].LN.LK[ILMV]	1	-23.04	[DLN]L.EL..E[EQ]	1	-20.75

L..SRGF	1	-19.77	[AG].HF[ILMV]GD	1	-24.32	[EQ]..P..[FHWY]P.E	1	-19.33

REPDP	1	-21.25	MLQ[QN]...[ILMV][KR]	1	-23.90	QL...N.L.[KMR][DLN]	1	-23.32

G.MFW	1	-16.96	[AG]KHF.G.G	1	-25.89	A[FHWY].[FHWY]E...EN	1	-24.56

L.G..VVP..S	1	-24.86	HAESGEYGL	1	-44.51	M[FHWY].[EQ][FHWY].D[ITV]	1	-23.90

MDHSNY	1	-28.88	[KR][ILMV].P.[ILMV][ST]..[FY]	1	-21.32	[ITV]..G[ITV].T.[ITV].V	1	-22.12

VL.G..TNI	1	-25.03	[AG][ST].D..GP	1	-18.86	[KMR]Y...N.V[CS]	1	-19.41

L..A..V.SS	1	-18.77	G...M.C..I	1	-16.47	[FHWY]..KG.[ITV].R[ITV]	1	-23.29

The patterns in Table 2 follow certain common conventions of regular expressions; specifically the dot (".") stands for any of the 20 amino acids and residues in square brackets belong to the same character class and are considered equivalent. Underlined amino acids constitute the residue where the *cis *peptide bond is detected within the pattern; it should be noted that some patterns do not capture the *cis *peptide bond itself but rather a sequential patterns in its neighborhood, therefore some patterns might have no underlined residues at all.

**Figure 4:**
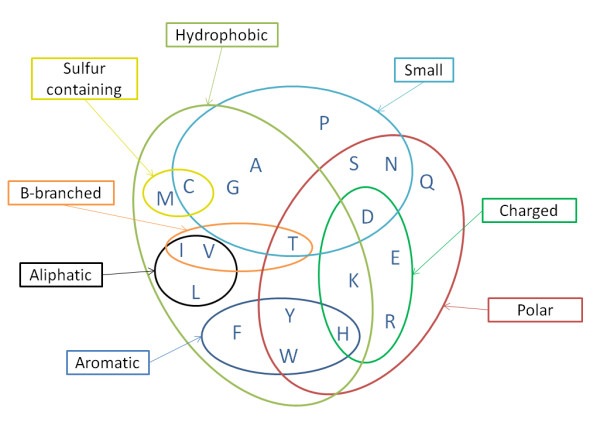
**Groupings of amino acids based on common physicochemical properties**.

From Table 2 we observe that all patterns in the top 20 list have yielded quite high values for *sc_score*, but the situation is also very similar for the entire list of retained associations. Especially for the cases where equivalency sets are employed all reported scores are even above 0.99, indicating that certain chemical and structural properties of the amino acids facilitate the discrimination between the two conformations. Such high score values denote high correlation of the maintained patterns with *cis *regions and low correlation with *trans *regions; this is also expressed by the values of FDR which are quite low for all types of patterns discovery and much lower when equivalency sets are employed. Another important observation is that certain patterns appear to be common, either unaltered or with slight variations, in all three types of pattern discovery; some example patterns of this kind are "KPGKGRRK", "EDGTKEPLL", "HAESGEYGL" and "ADEAT" which are all among the top 20 highest scoring patterns thus constituting good descriptors of *cis *formations. In order to facilitate the discussion that follows, we provide an overview of the amino acids' distribution among the most important physicochemical properties (Figure 4).

From the assessment of the retained patterns (partially shown in Table 2) several important corollaries can be deduced, some of which have been previously reported, subsequently supporting the rest of the assertions. For this purpose we provide in Table 3 the distribution of the amino acids among the retained patterns for each type of patterns discovery. In order to calculate the individual frequencies we have divided the occurrences of each residue by the total number of literals (i.e. all characters and character classes excluding wild characters). The frequencies of the character classes are also shown in the lower part of Table 3.

**Table 3:** Frequencies of occurrence for each residues and character class in the retained patterns.

	Amino acid frequencies (%)		
	**Exact pattern discovery**	**Chemical equivalency set**	**Structural equivalency set**

**A (alanine)**	8	5	6

**R (arginine)**	3	2	2

**N (aspargine)**	4	3	3

**D (aspartic acid)**	6	4	4

**C (cysteine)**	1	1	1

**E (glutamic acid)**	5	4	3

**Q (glutamine)**	2	1	2

**G (glycine)**	13	9	10

**H (histidine)**	3	2	2

**I (isoleucine)**	5	3	3

**L (leucine)**	9	6	6

**K (lysine)**	4	3	3

**M (methionine)**	2	1	1

**F (phenylalanine)**	4	3	2

**P (proline)**	6	4	4

**S (serine)**	7	5	5

**T (threonine)**	6	3	3

**W (tryptophan)**	2	2	1

**Y (tyrosine)**	4	3	2

**V (valine)**	7	4	4

**AG/ITV**	-	6	10

**DE/DLN**	-	3	7

**FY/KMR**	-	3	5

**QN/EQ**	-	2	2

**ILMV/FHWY**	-	12	7

**ST/CS**	-	5	2

**KR/-**	-	3	-

Regarding the column with the exact pattern discovery we observe that the obtained patterns are replete mainly with glycine, but also show a considerable preference towards leucine and alanine as well as serine and valine. The situation is quite similar for the other two types of pattern discovery when the frequencies of individual amino acids are considered, with proline, aspartic acid and glutamic acid yielding more or less the same frequency of occurrence as valine. These residues have been previously found to be prevalent in the neighborhood of *cis*-nonPro bonds, especially glycine and alanine which are also proven to be quite prevalent here as well [24]. Furthermore, it should be noted that same as with frequent residues, the most infrequent ones in the retained patterns are also the same for all types of pattern discovery. Specifically, cysteine exhibits the smallest representation (i.e. 1% in all types of pattern discovery) and the residues that follow are methionine, glutamine and tryptophan, however, not necessarily in this order for all types of pattern discovery. It is interesting that all four most frequent residues are small in terms of volume thus facilitating the formation of a *cis *bond by offering the minimum steric resistance. Moreover, we observe that glycine, alanine and valine are also hydrophobic residues, therefore conjecturing considerable preference of *cis *regions towards small and hydrophobic residues. However, cysteine is an exception to this corollary being a small hydrophobic residue as well, but hardly occurring in *cis *regions. A possible explanation lies probably in the sulfur atom that discriminates cysteine from the other three residues and is involved in the formation of the sulfhydryl group which is very reactive. Besides cysteine, methionine also contains sulfur in its side chain (and are both among the most infrequent amino acids), thus indicating the potentially unfavorable role of the sulfur atom towards the *cis*-nonPro peptide bond formation. Furthermore, glutamine and tryptophan that are rarely observed in the retained patterns are both polar and non-small residues, thus showing a negative association of *cis *regions and sizeable polar residues.

In the case of chemical equivalency set, when the character classes are also considered, [ILMV] has the highest frequency of occurrence and [AG] follows; on the contrary [QN] exhibits one of the smallest frequencies. Judging from the prevalent character class [ILMV] and more specifically from residues isoleucine, leucine and valine we observe a high propensity towards aliphatic residues. Character class [AG] appears with high frequency, which is in accordance with the previous observation for the individual residues alanine and glycine, and is further reinforced by the abundant occurrence of their combination as a character class. As for the structural equivalency set, character class [ITV] is the most frequent along with glycine; [DLN] and [FHWY] also show considerable representation in the retained patterns. The frequent occurrence of [ITV] character class shows a significant propensity of *cis *regions towards b-branched residues, which has also been reported in [24], especially in positions preceding the *cis *bond in order to stabilize the formation. Pal *et al*. [24] have also reported high propensity of short polar residues (serine, aspargine and aspartic acid) in *cis *regions which is only partially verified by our set of patterns. Specifically, if individual amino acids are considered, serine is the only abundant residue in the retained list of patterns, whereas aspartic acid and aspargine rank 8^th^ and 13^th^, respectively, for the case of exact pattern discovery, and in similar positions for the other two types of pattern discovery. However, this apparent discrepancy is justified by the high frequency of the [DLN] character class which accounts for several occurrences of aspargine and aspartic acid. Another frequent character class is [FHWY] featuring aromatic residues; it is worth noticing that even though the character class itself is quite abundant, all the individual residues are scarcely observed in any type of pattern discovery. Hence, there is a clear association between aromatic residues as a whole and *cis *regions which contribute towards the stabilization of the *cis *peptide bond [25], same as in *cis*-Pro bonds [24]. This preponderance for aromatic residues can be attributed to their ability to interact with adjacent side-chains via their π electron system [25]. Regarding the other common amino acid groupings, such as charged, we cannot deduce any significant association either with frequent residues or with infrequent ones; the charge does not seem to affect significantly the configuration of the peptide bond, at least not as a part of its immediate neighborhood, possibly, there exists such an influence originating from amino acids close in space but not necessarily close in sequence.

We have also assessed the distribution of *cis*-nonPro bonds across the 20 amino acids, in order to identify potential propensities towards specific residues; the respective frequencies are shown in Table 4 along with the frequencies for the residues preceding a *cis-*-nonPro formation.

**Table 4:** Frequencies of each residue occupying the position that *cis*-nonPro peptide bond occurs and the preceding one.

	Residues with *cis *bond	Preceding residue	Frequency of residues with *cis *bond (%)	Frequency of preceding residue (%)
**A**	23	22	7	7

**R**	**15**	**7**	**5**	**2**

**N**	15	14	5	4

**D**	26	23	8	7

**C**	5	4	2	1

**E**	27	20	8	6

**Q**	9	14	3	4

**G**	52	62	16	19

**H**	7	7	2	2

**I**	7	6	2	2

**L**	9	11	3	3

**K**	17	16	5	5

**M**	4	5	1	2

**F**	15	12	5	4

**P**	0	24	0	8

**S**	**28**	**13**	**9**	**4**

**T**	**22**	**12**	**7**	**4**

**W**	**2**	**16**	**1**	**5**

**Y**	16	14	5	4

**V**	19	16	6	5

First, we observe considerable propensity of the residue with the *cis *peptide bond towards certain amino acids, particularly for glycine [26], but also for serine, aspartic acid and glutamic acid. The high occurrence observed for aspartic acid and glutamic acid denotes a prevalence of the position with the *cis *bond towards negatively charged amino acids, similarly with the observations made in the retained patterns. Slightly reduced propensity is observed for alanine and threonine. Moreover, there are certain residues that very rarely participate in the formation of a *cis*-nonPro bond; such residues are methionine and tryptophan, as well as cysteine, histidine and isoleucine. All residues except maybe the case of glutamic and aspartic acid exhibited similar frequencies as well in the maintained patterns. It should be mentioned that the frequency distribution of certain amino acids in the position of the *cis *bond and the preceding one are very uneven; those residues appear in bold in Table 4 and are arginine, glutamine, serine, threonine, and most importantly tryptophan, which very rarely bears a *cis *bond but exhibits a considerable propensity in the preceding position. Clearly proline is excluded from this remark as the dataset contains only *cis*-nonPro peptide bonds and therefore the high difference in frequencies is biased.

### Functional implications

**Figure 5:**
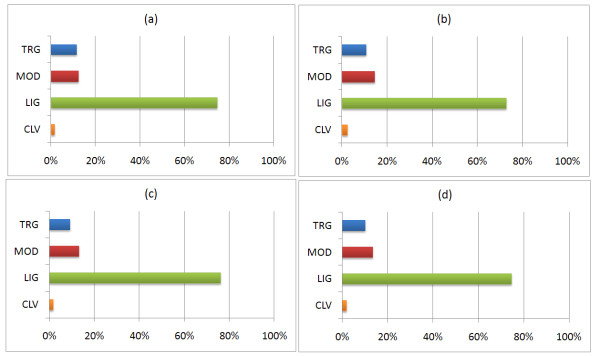
**Functional associations of *cis*-nonPro peptide bonds using: a) exact pattern discovery, b) pattern discovery with chemical equivalency set, c) pattern discovery with structural equivalency set and d) overall results from all types of pattern discovery**.

The obtained patterns are compared with the patterns of the ELM resource [15], in order to rediscover known functional implications of patterns associated with *cis*-nonPro regions but also identify new ones. All patterns extracted using each type of pattern discovery (i.e. exact pattern discovery, pattern discovery using chemical and structural equivalency set) have been compared with the patterns deposited in ELM using CompariMotif algorithm [22], and the respective results are shown in Figure 5; each bar contains the frequency towards each functional class of the ELM. The vertical axes of the plots contain the four major functional classes of the ELM repository, namely localization/targeting (TRG), post-translational modifications (MOD), binding/ligand (LIG) and cleavage (CLV). The complete list of the specific functional classes assigned to each pattern is available through the website.

We observe that the distribution of the retained patterns in the ELM functional classes is quite similar for all types of pattern discovery; specifically the LIG class is reported in approximately 80% of the patterns, TRG and MOD are almost equally reported for accounting for almost 15% of the patterns each, and CLV follows with less than 5%. Those values reported by Comparimotif are compared via chi-square test with the respective values expected by chance, in order to verify that the reported propensity is statistically significant. The expected values refer to the representation of each functional class within the ELM and are specifically, for TRG: 13%, MOD: 25%, LIG: 54% and CLV: 7%. Using a relatively strict significance level of 0.01 [27], we conjecture statistically significant propensity towards the LIG functional class; using all types of pattern discovery. A significant association is also reported for the MOD class, however only when exact pattern discovery is employed, subsequently diminishing the credibility of the yielded association. The significant propensity towards LIG is further reinforced by [28] where *cis*-nonPro peptide bonds were reported to be intimately involved in ligand binding as well as the positioning of catalytic residues. This prevalence has been attributed to the greater precision offered by the strained *cis*-nonPro formation which in turn is necessary for ligand binding and catalysis. Moreover, it has been elsewhere reported that *cis*-nonPro formations are located at or near active sites or functional regions of the protein molecule which are closely related to ligand/binding activities [3,24,25]; thus explaining the high propensity towards the LIG class.

The smaller propensity that is observed towards MOD is partially justified by the relevant literature, where sporadic associations have been proposed, with respect to carbohydrate binding or carbohydrate processing proteins [25]. Nevertheless, modification sites usually have ligand activity [29] which in turn has been proven to be inherently related to *cis*-nonPro regions.

In the next step we follow a somewhat reverse analysis in order to verify if the functional class attached to a set of patterns actually falls within the function of the protein sequences from which the patterns have been extracted. We perform this procedure for a subset of representative functional classes, specifically for a few among the top scoring ones from each type of pattern discovery. In order to identify those functional classes we first group the patterns according to the ELM functional class that they recovered and for each class we calculate the average *CM_score *(based on the output of CompariMotif). For the patterns associated with the top scoring classes we retrieve the sequences from which they have been extracted and check if the function of those sequences matches the respective ELM function. For the comparison we employ the GO terms retrieved from the ELM website and UniProt [30] for the functional classes and the protein sequences, respectively. The results obtained are shown in Table 5.

**Table 5:** Functional verification between ELM functional classes and the respective associated sequences.

ELM		Sequences	
**Functional class**	**GO category**	**PDB id**	**GO category**

LIG_14-3-3_2	Binding	2O34A, 2FPHX, 1NAR0, 2DDXA	Binding, catalytic activity

LIG_PP1	Binding, enzyme regulator activity	2HHPA, 2HFKA, 1D3YA, 1OI7A, 1ZY7A, 2B18A, 2F2HA, 2GZQA	Binding, catalytic activity, transporter activity, transcription regulator activity

LIG_PP2B_1	Binding, catalytic activity	1ES5A, 1T8TA, 1EZWA, 1V5VA	Catalytic activity

LIG_MAPK_1	Binding	1B43A, 1NSZA, 2GAKA, 2O34A, 1VPDA, 2NQTA	Binding, catalytic activity,

LIG_SCF-TrCP1_1	Binding, catalytic activity	1B43A, 1Y8TA, 1M4LA, 2IDLA	Binding, catalytic activity

LIG_14-3-3_3	Binding	2IDLA, 2HHPA, 2O34A, 1UG6A, 2C0HA, 1B12A, 1O54A, 2FPHX, 1GNLA, 2FMPA, 1D3GA, 2ES4D, 2DXQA, 2A67A, 2G0WA, 1PO5A, 1Q74A, 1CNV0, 1JNDA, 1KMVA, 2JG0A, 7A3HA	Binding, catalytic activity

LIG_EH1_1	Binding	1B12A, 2C0HA, 1WDPA, 2CK3H, 1O54A, 2HHPA, 1R5YA, 2D0OA, 1USGA, 2DG1A, 1O2DA, 1YACA, 1RYIA, 1VZIA, 1NAR0, 2DSKA, 1NTHA, 2AFWA, 2C61A, 1D3GA, 1NNWA, 2NX9A, 1X13A, 2A67A, 1C8XA, 2AEEA, 1YDYA, 2GAKA	Binding, catalytic activity

LIG_BRCT_BRCA1_1	Binding	1FSGA, 1OQ1A, 2FPQA, 1ZMTA, 2AZ4A, 1NOFA, 1UEKA, 2HSIA	Binding, catalytic activity

LIG_NRBOX	Binding	1M4LA, 2NT0A, 2NX9A, 1VLRA, 2DJFA, 1DQPA, 1G2QA, 1J2RA, 1NF9A, 1ITXA, 2JE8A, 1B25A, 1CNV0, 1NAR0, 2DSKA, 2CK3H, 2IACA, 1FSGA, 1VJPA, 1NTHA, 2HSIA	Binding, catalytic activity

LIG_CORNRBOX	Binding	1O54A, 2C61A, 1C8XA, 1LBVA	Binding, catalytic activity, transporter activity

First of all, we observe that all top scoring functional classes are of the LIG type and therefore involve binding, as shown from the GO category as well. In addition, some of them are also related to catalytic activities, such as LIG_PP1, LIG_PP2B_1 and LIG_SCF-TrCP1_1. This high propensity towards binding related functionalities is in accordance with the literature where there has been identified a close relation between *cis*-nonPro formations and binding/ligand functions, and especially carbohydrate binding [25]. Indeed, among the protein sequences of the top scoring functional classes there are some sequences specifically associated with carbohydrate binding (PDB ids: 1NSZ, 7A3H, 1ITX, 2JE8). Moreover, we observe complete overlap between the GO category of the ELM functional classes and the GO category assigned to the sequences retrieved, where in all cases the binding functionality has been assigned, and in some cases catalytic activity, as well as a few other functionalities such as transporter activity and transcription regulator activity. Nevertheless, in all cases both binding and catalytic activity overlap between the ELM categories and the GO category of the respective sequences.

### Peptide bond classification

In the last step of the proposed methodological analysis we employ the extracted patterns in order to perform pattern-based classification of amide peptide bonds. As mentioned previously, for evaluation purposes we extract patterns from the 2/3 of the *CNP *dataset, and assess their predictive potential against 5 randomly assembled independent test sets. The classification function is rather simple and uses all extracted patterns in order to search for matches against the regions of the test set. The regions yielding at least one match are marked as *cis*, whereas regions with zero matches are assigned as *trans*. The performance is quantified by measuring sensitivity, specificity and accuracy over the 5 test sets and averaging the results (Table 6). Sensitivity and specificity denote the fraction of correctly assigned *cis*-nonPro and *trans*-nonPro regions, respectively, and accuracy is a measure of the overall correctness.

**Table 6:** Comparison of available methodologies for the classification of amide peptide bonds.

Author	Method	Sensitivity (%)	Specificity (%)	Accuracy (%)
Pahlke *et al*. [8]	Chou-Fasman parameters	35	97	66

Exarchos *et al*. [9]	SVM classifier	77	65	71

Current work	exact pattern discovery	45	54	49
	
	chemical equivalency set	76	58	67
	
	structural equivalency set	77	63	70

Even though in the literature several methods have been proposed for predicting the peptide bond conformation of proline residues, only [8] and [9] aim at discriminating between *cis*-nonPro and *trans*-nonPro bonds. Table 6 presents a comparison between the proposed methodological analysis and the ones reported in the literature. We observe that the proposed methodological analysis yields quite encouraging results, especially when the chemical and structural equivalency sets are considered. Besides its simplicity, the proposed pattern-based classification scheme features transparency during its flow of operation, by reporting the sequential pattern designating and dictating the conformation of the specific peptide bond assigned as *cis*-nonPro.

## Conclusions

We presented a methodological analysis for extracting and annotating protein patterns. Specifically, the study focused on regions surrounding the scarce, yet highly important *cis*-nonPro formations. The elicitation of regular expression-type patterns from these regions has been motivated by the limited amount of available *cis *amide peptide bonds and to this end aims to facilitate the extrapolation and generalization of gained knowledge about these formations, in a more systematic manner. The resulting list of patterns contains approximately 200 patterns achieving 100% coverage of *cis*-nonPro bonds and FDR around 0.03% for *trans*-nonPro bonds. The retained patterns are subsequently evaluated in terms of their predictive potential towards discriminating between *cis*-nonPro and *trans*-nonPro peptide bonds, yielding quite encouraging results. Especially favorable for predictive purposes are the pattern sets obtained using the chemical and structural equivalencies among amino acids. Our findings confirmed that regions containing *cis*-nonPro peptide bonds appear replete with glycine as well as leucine and alanine, which facilitate the formation of the *cis *bond given their refined volume; a considerable propensity was also observed towards aromatic residues which possibly act as a wrench for the stabilization of the adjacent bond. Among the retained patterns we observed lack of cysteine and methionine which can be attributed to the sulfur atom and subsequently the highly reactive sulfhydryl group. Moreover, regarding the functional associations of *cis*-nonPro bonds we observed a high prevalence for ligand/binding sites.

## Availability

For reproducibility reasons, all datasets and algorithms employed in this work, as well as extensive results and links to relevant resources, have been deposited and are available in the following URL: http://sites.google.com/site/cnppatterns/.

## Authors' contributions

KPE conceived, designed and implemented the study, working towards his PhD thesis. TPE and CP provided valuable comments and discussions. GR has contributed to the implementation of the scripts and algorithms. DIF supervised the study and provided substantial advice and guidance during all phases. All authors have read and approved the final manuscript.
